# Food availability of glucose and fat, but not fructose, increased in the US between 1970 and 2009: analysis of the USDA food availability data system

**DOI:** 10.1186/1475-2891-12-130

**Published:** 2013-09-23

**Authors:** Trevor J Carden, Timothy P Carr

**Affiliations:** 1 Department of Nutrition and Health Sciences, University of Nebraska, 68583-0806 Lincoln, Nebraska, USA

**Keywords:** Fructose, High fructose corn syrup, Food availability, USDA database

## Abstract

**Background:**

Obesity rates in the United States have risen consistently over the last four decades, increasing from about 13% of the population in 1970 to more than 34% in 2009. Dietary fructose has been blamed as a possible contributor to the obesity increase, although the consumption pattern of fructose and other key nutrients during this 40 year period remains a topic of debate. Therefore, we analyzed the USDA Loss-Adjusted Food Availability Database in combination with the USDA Nutrient Database for Standard Reference (Release 24) to determine whether fructose consumption in the US has increased sufficiently to be a casual factor in the rise in obesity prevalence.

**Methods:**

Per capita loss-adjusted food availability data for 132 individual food items were compiled and analyzed. Nutrient profiles for each of these foods were used to determine the availability of energy as well as macronutrients and monosaccharides during the years 1970-2009. The percent change in energy from food groups and individual nutrients was determined by using the year 1970 as the baseline and area-under-the-curve analysis of food trends.

**Results:**

Our findings indicate that during this 40 year period the percent change in total energy availability increased 10.7%, but that the net change in total fructose availability was 0%. Energy available from total glucose (from all digestible food sources) increased 13.0%. Furthermore, glucose availability was more than 3-times greater than fructose. Energy available from protein, carbohydrate and fat increased 4.7%, 9.8% and 14.6%, respectively.

**Conclusions:**

These data suggest that total fructose availability in the US did not increase between 1970 and 2009 and, thus, was unlikely to have been a unique causal factor in the increased obesity prevalence. We conclude that increased total energy intake, due to increased availability of foods providing glucose (primarily as starch in grains) and fat, to be a significant contributor to increased obesity in the US.

## Background

Obesity is a persistent public health crisis with a myriad of health and economic consequences. Obesity rates in the United States have risen consistently over the last four decades, increasing from about 13% of the population in 1970 to more than 34% in 2009 [1]. Consequently, researchers have focused increasingly more effort towards uncovering the causes of obesity in order to determine effective methods for reversing the condition.

Within the last decade, the unique metabolic handling of fructose compared to glucose has been highlighted, leading to increased suspicion of a contributory and possibly even causal role for fructose in the US obesity epidemic [2,3]. High fructose corn syrup (HFCS) contributes significantly to total fructose consumption and, consequently, has inherited a reputation as a causal factor of obesity as well [3,4]. But HFCS and fructose are not synonymous and the distinction between them is often lost. Indeed, conclusions about fructose *per se* are often directly applied to HFCS resulting in the two sweeteners being equally implicated in obesity [3]. Because of the misunderstood connection between HFCS and fructose, misconceptions about fructose consumption trends have multiplied as well. For instance, it is often stated that total fructose consumption has greatly increased since 1970, when in reality it is HFCS usage that has greatly increased, while fructose usage *per se* has stayed relatively stable with only small changes.

Although HFCS and sucrose are comprised of similar ratios of fructose to glucose, the latter sugars exist as disaccharides in sucrose and monosaccharides in HFCS. This difference has been explored as a possible explanation for why the replacement of sucrose with HFCS in the food supply that has taken place since 1967 may have led to obesity. Fructose and glucose consumed together as monosaccharides have been alleged to be sweeter and to yield a greater gastric osmotic pressure compared to the disaccharide sucrose. Such characteristics have been invoked to explain how HFCS may elicit uniquely negative effects compared to sucrose [5]. Claims regarding increased osmotic pressure from HFCS, however, have been little more than editorial speculation without the offer of evidence or a physiological rationale, and the idea that HFCS is sweeter than sucrose is contrary to empirically and mathematically derived sweetness values [6,7]. Furthermore, although there is a paucity of studies comparing the metabolic effects of HFCS and sucrose, the studies that have been conducted so far, especially human trials, do not support the existence of significant differences between the two [8-10]. While some have argued that the use of HFCS should be restricted [4,11], others have pointed out the lack of evidence to ban or otherwise restrict the use of HFCS [12-14].

The aim of this study was to determine whether total dietary fructose is likely to have contributed to obesity to the degree that is alleged and, if not, what other factors likely did. When considering the issue of rising prevalence of obesity from the perspective of dietary intake trends, as this article does, fructose could be a contributing factor if its intake increased sufficiently to have influenced an upward trend in total energy intake and positive energy balance. Thus, we examined the change over a 40 year period of total fructose availability, as well as the trends of other important nutrients and nutrient classes, in order to assess which components of the American diet have most likely contributed to the obesity epidemic. To accomplish this goal, US per capita loss-adjusted food availability data from the United States Department of Agriculture (USDA) were collected and used as a proxy for food consumption to estimate and analyze trends of the US food and nutrient consumption from 1970-2009.

## Methods

### USDA data collection

Food availability data from 1970-2009 were collected from the USDA Economic Research Service, loss-adjusted food availability database [15]. Food availability data were collected as g/d per capita of 132 individual food items (see Additional file 1). Nutrient composition profiles were collected for total fat, protein, carbohydrate, lactose, maltose, sucrose, fructose, glucose, galactose and lactose using the USDA Nutrient Database for Standard Reference, Release 24 [16]. The per capita quantity of loss-adjusted food availability of each food item (g/d) was multiplied by each nutrient (g of nutrient/g of edible food) in the nutrient profile to arrive at a total availability (g/d and kcal/d) of each nutrient, which were then summed for all foods to determine the total daily availability per capita of each nutrient in the US food supply.

Because the USDA nutrient database is lacking in monosaccharide and disaccharide composition for many foods, the UK Composition of Foods Integrated Dataset (CoFIDS) [17] was used to obtain the missing information for 20 of the 132 food items (see Additional file 1). The monosaccharide and disaccharide breakdown of foods compared between the USDA nutrient database and CoFIDS are often quite disparate. Therefore in order to integrate the CoFIDS data with the USDA data, the proportion of each monosaccharide or disaccharide of total sugar as calculated from CoFIDS was applied to the total sugar content of each food as provided by the USDA database rather than directly applying the monosaccharide masses obtained from CoFIDS.

Data in the tables and figures regarding fructose are presented as all fructose consumed in its monosaccharide form plus all fructose consumed as sucrose. Likewise, glucose data represent glucose available as a monosaccharide plus all glucose available from sucrose, lactose, maltose and starch. In this way, data presented herein represent the total amount of fructose and glucose available for intestinal absorption irrespective of the food or chemical form available for consumption.

### NHANES data collection and adjustment for obesity

National Health and Nutrition Examination Surveys (NHANES) contain, in part, self-reported dietary intake data. These data are useful for assessing the validity of USDA food availability data. However, self-reporters of food intake such as those surveyed in NHANES are known to be frequent and dramatic under-reporters of energy intake in particular [18,19]. In addition, two major determinates of under-reporting of energy intake are gender and body mass; women under-report to a greater extent than men, and obese men and women under-report more than their lean counterparts [18,20]. Because obese individuals under-report to a greater extent than lean individuals, and because obesity rates have more than doubled since 1970, it is reasonable to expect that under-reporting of energy intake in NHANES has also increased. As a result, energy trends as assessed by NHANES do not fully capture the rise in energy consumption.

To adjust energy intake for under-reporting, kcal/d, BMI, gender and sample weights were collected from NHANES I-III as well as the Continuous NHANES by utilizing Statistical Analysis Software (SAS Institute, Cary, NC) data extraction procedures developed and distributed by the National Center for Health Statistics. Only participants with data for all four categories were included in the analysis. Data were sorted by gender, and gender-specific correction factors derived from literature values [20] estimating the under-reporting tendencies of obese males and females were multiplied by the self-reported kilocalorie intakes of each participant with a BMI ≥ 30 kg/m^2^. A numeric value known as a sample weight, which was derived by the National Center for Health Statistics for each participant within an NHANES dataset to correct for oversampling of minority populations, was multiplied by their reported caloric intake and divided by the sum of all sample weights. This weighted calorie intake was then summed across all participants, thus creating a weighted, obesity-adjusted, average calorie intake that is representative of the entire non-institutionalized US population.

### Area under the curve (AUC)

Assessing food trends by comparing only the first and last years or the highest and lowest points in the trend can be potentially misleading, so it is important to determine the trend’s cumulative change over time. Therefore, we developed a means of comparing the trend of food availability for the entire period of 1970-2009 by establishing a 1970 “baseline” AUC assuming that food availability did not change (flat line) from 1970 to 2009. Then an “actual” AUC was calculated using the actual data from 1970-2009. The difference between the two AUC measures was used to determine a percent cumulative change for each trend. The difference between the two AUC measurements was used to determine a percent cumulative change for each food category, macronutrient, and total energy.

Per capita food availability was converted from g/d to kcal/d as described above and the overall cumulative change was determined as follows:

$$\%\;\mathrm{Cumulative}\;\mathrm{Change}=\frac{\left(\mathrm{Actual}\;\mathrm{AUC}-\mathrm{Baseline}\;\mathrm{AUC}\right)}{\mathrm{Baseline}\;\mathrm{AUC}}\;\times \;100$$

## Results

### Macronutrient availability per capita

**Figure 1:**
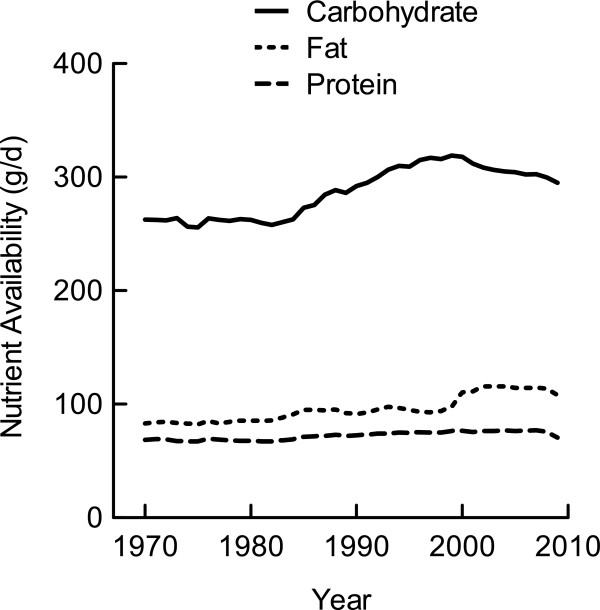
**Macronutrient availability per capita in the US.** Data were derived from the USDA Food Availability Database [15].

The availability of the major macronutrient classes increased in the US during the period between 1970 and 2009 (Figure 1). The amount of carbohydrate available for consumption increased from 262.7 g/d in 1970 to its highest at 318.9 g/d in 1999, then it decreased to 295.0 g/d by 2009. The maximum range of carbohydrate availability (i.e., lowest value compared to highest value) during the 1970-2009 period was 63.8 g/d, or 255.2 kcal/d. The cumulative change in carbohydrate-derived energy, according to the trend change in AUC, was 9.8% above the 1970 baseline (Table 1).

**Table 1:** Cumulative change in nutrient energy availability per capita, 1970-2009

				**Carbohydrate**		
	**Total energy**	**Protein**	**Fat**	**Total**	**Glucose**	**Fructose**
Energy available in 1970, kcal/d^1^	2143	276	751	1050	773	253
Net energy accumulation during 1970-2009, kcal^2^	8986	486	4268	3915	3915	−0.7
% change^3^	10.7	4.7	14.6	9.8	13.0	0.0

^1^Values for 1970 were used to establish a constant baseline AUC for the entire period between 1970 and 2009.

^2^Values represent the actual AUC trends derived from annual data.

^3^Values were calculated from actual AUC compared to baseline AUC.

Dietary fat availability increased from 82.2 g/d in 1970 to 97.5 g/d in 1999 (Figure 1). It then rapidly increased to 115.7 g/d from 2000 to 2004 remaining elevated at 113.7 g/d through 2008 until 2009 when it decreased to 107.9 g/d. The range of fat availability during the 1970-2009 period was 33.5 g/d or 301.5 kcal/d. The cumulative change in fat-derived energy was 14.6% compared to the 1970 baseline (Table 1).

Dietary protein availability also increased from 67.1 g/d in 1970 to 77.0 g/d in 2009, with a range of 9.8 g/d or 39.2 kcal/d (Figure 1). The cumulative change in protein-derived energy was 4.7% above the 1970 baseline (Table 1).

### Fructose and glucose availability per capita

**Figure 2:**
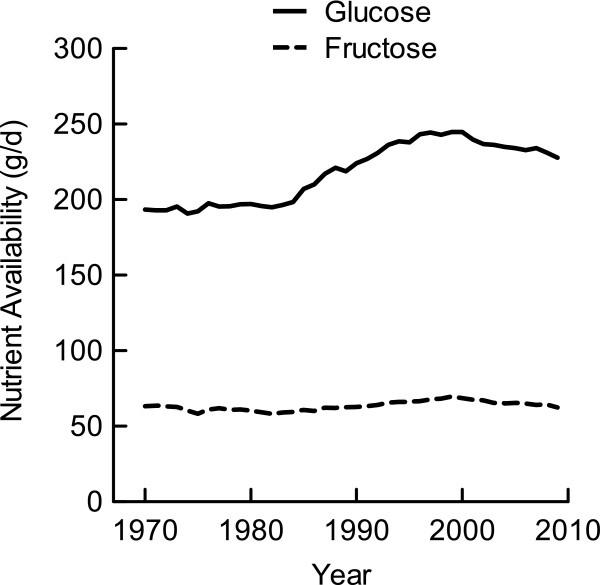
**Glucose and fructose availability per capita in the US from all food sources.** Food category data were derived from the USDA Food Availability Database [15]. The saccharide composition of foods was determined using the USDA Nutrient Database for Standard Reference, Release 24 [16] and the UK Composition of Foods Integrated [17].

From 1970 to 1984, total glucose availability from all food sources varied slightly from 193.4 g/d to 198.4 g/d; however, from 1985 to 2000 it increased to its peak at 244.8 g/d and then decreased to 227.8 g/d in 2009 (Figure 2). The range of glucose availability from 1970 to 2009 was 54.1 g/d or 216 kcal/d. The cumulative change in glucose-derived energy during the 1970-2009 period increased 13.0% compared to the 1970 baseline (Table 1). The net energy accumulation of glucose and total carbohydrate are essentially the same because of the trivial change in fructose accumulation (-0.7 kcal/d) and galactose accumulation (0.6 kcal/d; galactose data not shown in table).

Total fructose availability from all food sources remained comparatively steady during the 1970-2009 period (Figure 2). Fructose availability was 63.2 g/d in 1970; it decreased to its lowest point at 58.2 g/d in 1982, increased to its highest point at 69.5 g/d in 1999, then decreased to 62.4 g/d in 2009. The difference between the highest and lowest values was a modest 11.3 g/d (or 45 kcal/d), resulting in no cumulative change in fructose-derived energy during the 1970-2009 period (Table 1).

### Energy availability per capita

**Figure 3:**
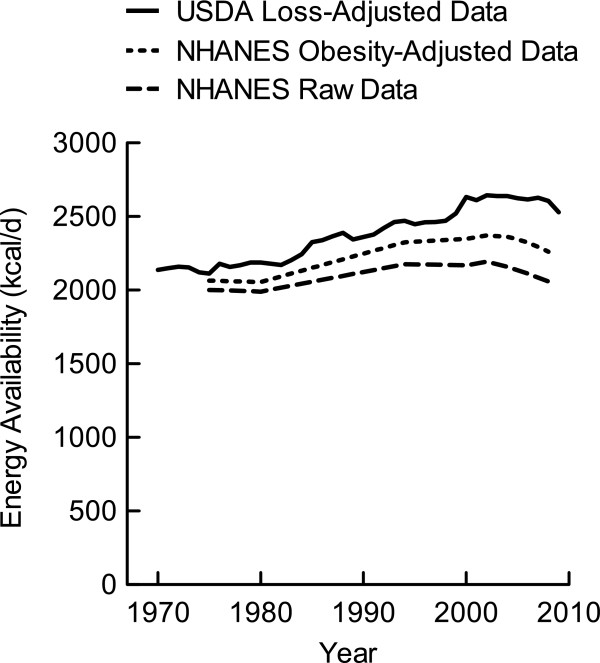
**Total energy availability per capita in the US.** Data were derived from the USDA Food Availability Database [15] and the NHANES I-III and the Continuous NHANES from 1999-2008.

Total available energy in 1970 was 2137 kcal/d. Between 1970 and 1980, energy availability increased 2.4% (52 kcal/d). Between 1980 and 1990, energy availability increased 7.9% (173 kcal/d), and between 1990 and 2000 it increased 11.5% (271 kcal/d). Energy availability plateaued from 2000 to 2008 where it remained between 2644 and 2606 kcal/d before decreasing to 2530 g/d in 2009 (Figure 3). Over the 40 years that this analysis covered, the range was 532 kcal/d and the cumulative change in total energy increased 10.7% above the 1970 baseline (Table 1).

In an effort to validate our analysis of the USDA loss-adjusted food availability data, we also analyzed total energy trends across the same period of time using data from NHANES I-III as well as the Continuous NHANES from 1999-2008 (Figure 3). Total energy trends from both the USDA and NHANES databases indicated increased energy availability between 1980 and 2000. Because obese survey participants more frequently and dramatically underestimate their food intake [21,22], we adjusted the NHANES data for participants whose BMI indicated obesity (≥ 30 kg/m^2^). The adjustment resulted in an NHANES energy trend that more closely matched the USDA trend in both slope and magnitude. Whether adjusted or unadjusted, the NHANES data paralleled the same trends as the USDA loss-adjusted data.

The contribution of nine food categories to total energy availability was analyzed from 1970-2009. The 1970 level of energy availability, net energy accumulation from 1970 to 2009, and the percent change for each food category are shown in Table 2. Of the major categories contributing energy to the diet, the largest percent change occurred in the grains and the fats/oils categories, increasing 24.2% and 25.3%, respectively. In contrast, the sweeteners category increased 1.3% over the 40 y period. Alcohol, fruits, vegetables, and nuts each demonstrated a net increase in energy contribution between 1970 and 2009, although these categories combined contributed < 15% of daily total energy.

**Table 2:** Cumulative change in food energy availability per capita, 1970-2009

	**Sweeteners**	**Grains**	**Fats/Oils**	**Meat**	**Dairy**	**Alcohol**	**Fruits**	**Vegetables**	**Nuts**
Energy available in 1970, kcal/d^1^	410	404	368	338	279	113	72.3	112	47.0
Net energy accumulation during 1970-2009, kcal^2^	212	3807	3641	−184	−440.1	1025	501	87.9	337
% change^3^	1.3	24.2	25.3	−1.4	−4.0	23.3	17.8	2.0	18.4

^1^Values for 1970 were used to establish a constant baseline AUC for the entire period between 1970 and 2009.

^2^Values represent the actual AUC trends derived from annual data.

^3^Values were calculated from actual AUC compared to baseline AUC.

### Sucrose and HFCS trends

**Figure 4:**
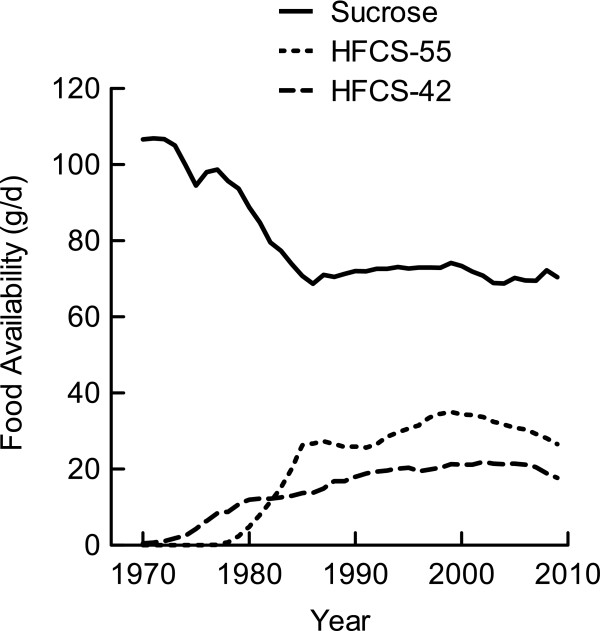
**Sucrose and HFCS availability per capita in the US.** Data were derived from the USDA Food Availability Database [15].

The caloric sweeteners category consisted primarily of sucrose, HFCS-55, and HFCS-42. The availability of sucrose decreased between 1970 and 1986, dropping from 106.6 g/d to 68.7 g/d, and remaining below 75 g/d through 2009 (Figure 4). In a reciprocal manner to sucrose, the availability of both types of HFCS (dry weight equivalent) increased since their introduction into the US food supply (Figure 4). HFCS-55 availability reached its highest point at 35.0 g/d in 1999 and decreased to 28.1 g/d in 2009. HFCS-42 availability reached its peak at 21.9 g/d in 2002.

**Figure 5:**
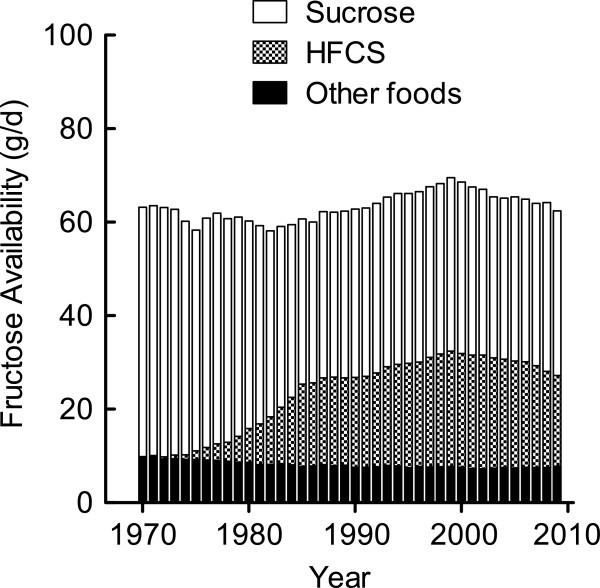
**Fructose availability per capita in the US from sucrose and HFCS.** Food category data were derived from the USDA Food Availability Database [15]. The fructose composition of foods was determined using the USDA Nutrient Database for Standard Reference, Release 24 [16] and the UK Composition of Foods Integrated [17].

The amount of fructose available from sucrose, HFCS, and all other foods is depicted in Figure 5. The data indicate that sucrose and HFCS, used as added sweeteners, are the major sources of fructose in the US diet. Other food sources of fructose include fruits, vegetables, honey, and other syrups.

## Discussion

Obesity is a physiological state characterized by excessive storage of triglyceride in adipose depots. Because accumulation of triglycerides in adipocytes requires a positive net energy supply, it follows logically that the greatest dietary contribution to obesity would be made by the dietary source or sources that supply the most energy. Therefore, this study was initiated to determine whether fructose could have provided sufficient energy to cause the dramatic rise in obesity since the early 1970s and, if not, what other explanations may exist. To achieve this goal, USDA loss-adjusted food availability data were collected to assess nutrient trends of the average, non-institutionalized American. The phrase “food availability” is often used interchangeably with the phrase “food disappearance,” highlighting the usability of these data as a proxy for food consumption. These data are therefore presented as an estimate of the amount of foods and nutrients consumed daily by the average American. The data do not provide information on specific demographic groups, but rather the entire U.S. population on a per capita basis. The analysis is strengthened by our ability to track trends over several decades using data collected annually. Furthermore, the correction of these data for typical losses such as plate waste and inedible portions allows for more accurate estimates and is a unique characteristic of this analysis as compared to other fructose consumption analyses that use unadjusted USDA data. An additional unique quality of this analysis is the consideration of the amount of individual monosaccharides and disaccharides (whether consumed as simple or complex carbohydrates) by applying the nutrient composition of each food to the mass of each food available for consumption.

The availability of carbohydrates in the US food supply has increased more than any other macronutrient since 1970. In 2009, 32.3 g/d more carbohydrate was available than in 1970, suggesting that carbohydrates alone contributed 129 kcal/d more in 2009 than 1970 with a range of 253 kcal/d. The net accumulation of carbohydrate across the study period as measured by AUC was 9.8%. It is important to ask how much of this increase may be attributed to fructose from all sources. Estimates of total fructose consumption have been reported using the unadjusted USDA food availability data [4] and survey data from the Nationwide Food Consumption Surveys, the Continuing Survey of Food Intake by Individuals, and the NHANES [23], with the conclusion that per capita fructose consumption increased 18% between 1970 and 2004. The purported increase was suggested as support for a causal role of fructose in obesity. In the present study using loss-adjusted data, we observed fluctuations in fructose availability that included periods of increase and decrease between 1970 and 2009, with an overall result of no net change in total fructose availability.

Several factors must be considered to properly interpret the biological significance of the fluctuations in fructose availability. First, we observed a maximum range of increase in total fructose availability of 11.3 g/d between 1982 and 1999, representing an increase of 45 kcal/d during this 18 y period. It is tempting to associate this increase with the greatest rise in obesity that was observed between 1980 and 2000. However, the increase of 45 kcal/d was minor compared to the increase in total glucose (49.8 g/d) and fat (11.9 g/d) availability that together accounted for an increase of 306 kcal/d during the same 18 y period. While our analysis did show an increase in total fructose availability from 1982 to 1999, emphasizing this change in isolation without considering the much larger changes in glucose, fat and total energy availability unduly magnifies and distorts the contribution of fructose to the rise in obesity. Previous reports that have attempted to link fructose consumption and obesity have not taken into account the comparatively large increases in glucose, fat and total energy availability that have occurred since 1970 [4,23]. Second, our analysis indicated a decrease in caloric sweeteners and total fructose availability from 1999 to 2009 while obesity trends continued to increase during the same period, illustrating a lack of association between fructose consumption and obesity. Sun et al. [24], using the NHANES 1999-2006 databases, also reported no positive association between fructose consumption and body mass index or waist circumference. The increase in fat availability since 1999 appears to account for the continued high level of total energy available. Third, using unadjusted USDA food availability data overestimates nutrient consumption by as much as 30-75% compared to USDA loss-adjusted data, as estimated by the difference between the raw and unadjusted numbers of foods with the greatest and smallest adjustments (data not shown). Our analysis using loss-adjusted data indicated that total fructose availability in 1970 and 2009 were nearly identical; the range of fructose availability was only 11.3 g/d over the 40 y period; and its net accumulation was effectively zero despite significant changes in the food systems that provide fructose, such as using HFCS as a substitute for sucrose in foods and beverages. During the same time, the prevalence of obesity in the US significantly increased, indicating that dietary fructose *per se* could not have played a quantitatively important role in the increased prevalence of obesity.

Despite the current findings showing a lack of association between total fructose availability and obesity prevalence, one should not regard fructose as a benign nutrient that can be consumed without consequence, particularly if over-consumed. Fructose is a lipogenic nutrient and metabolized differently than glucose [25]. Relative to glucose, excessive consumption of fructose (25% of total calories) was reported to elevate plasma concentrations of apoB, triglycerides, and LDL cholesterol [26]. Fructose may also elevate circulating levels of uric acid and multiple liver enzymes relative to glucose [27], although a recent meta-analysis of 21 trials failed to show a uric acid-increasing effect of isocaloric fructose intake [28]. In a study of adult men and women, dietary fructose relative to glucose elevated blood flow to regions of the brain that regulate appetite and also reduced circulating levels of satiety hormones [29]. These observations demonstrate unique features of dietary fructose that require further study, especially in the context of excess fructose and excess total calories [8-10,30-32]. The current study suggests that the unique lipogenic properties of fructose may have been of minor importance to the rise in obesity due to the small contribution of dietary fructose relative to glucose and total energy availability.

The present findings also cast doubt on the purported role of HFCS as a singly important dietary factor in promoting obesity. Despite increased usage of HFCS in the US food supply, no net change in total fructose availability occurred between 1970 and 2009 when analyzed using loss-adjusted data. A critical review of epidemiologic studies and randomized controlled trials failed to demonstrate a relationship between HFCS consumption and increased obesity prevalence [12]. Since the introduction of HFCS, their usage has been accompanied by decreased usage of sucrose. Unfortunately, no standard methodology or application of HFCS has been employed in human and animal studies, leading to inconsistent results [14]. Furthermore, the US diet contains products made with both HFCS-55 and HFCS-42, which when consumed in combination within the context of the entire diet, yields nearly identical availability of glucose and fructose. Studies that test only HFCS-55 in comparison to sucrose have a disproportionately higher dietary fructose-to-glucose ratio [30].

If fructose and HFCS are unlikely to have contributed to obesity in a direct manner, then what other dietary nutrients and food sources might be responsible for the increase in total energy between 1970 and 2009? Carbohydrate availability increased more than any other macronutrient. When calculated on a monosaccharide basis from all food sources, the most abundant carbohydrate was glucose. In 1970, there was approximately 3.1-times more glucose than fructose available in the food supply; in 2009, there was 3.6-times more glucose than fructose. The increase in glucose availability from 1970 to 2009--and the lack of an increase in fructose availability--was due to an increased availability of glucose-containing food sources other than caloric sweeteners. Indeed, our findings indicate that the grains category provided more energy and increased more than any other glucose source.

Dietary fats/oils availability also increased from 1970 to 2009. Starting in 1999, fats/oils availability increased sharply while carbohydrate availability began to decline. This apparent replacement of carbohydrate with fats/oils may have been due to increasing popularity of diets lower in carbohydrate and higher in fat, thus accounting for the greater net accumulation of energy from fat versus carbohydrate. Before this macronutrient shift in 1999, carbohydrate had accumulated to a greater extent than fats/oils regarding both mass and energy contribution. Therefore, it appears that from 1970 to 1999, carbohydrate made the greatest contribution to the increased energy availability of the US diet, whereas the fats/oils category was a more important contributor to the energy increase after 1999. The increased availability of the grains and fats/oils categories was further demonstrated by the AUC approach we employed to estimate net accumulation of energy in relation to 1970 food availabilities. This finding is consistent with other food trend studies using both NHANES and the Nationwide Food Consumption Survey in which total energy and total carbohydrate intake have increased since 1970 [33,34].

## Conclusions

Our analysis using USDA loss-adjusted food availability data indicated total energy availability in the US food supply increased 10.7% from 1970 to 2009. The food categories that increased the most during this time were grains and fats/oils, having increased 24.2% and 25.3%, respectively. Caloric sweeteners (including both sucrose and HFCS) increased a modest 1.3%. When expressed in terms of monosaccharides available for metabolic absorption, all carbohydrate food sources provided > 3-times more glucose than fructose. Moreover, total glucose availability increased 13.0% from 1970 to 2009, whereas total fructose availability did not change. Our findings indicate that fructose *per se* was not a unique causal factor in promoting obesity during 1970-2009. Rather, we conclude that increased total energy intake, due to increased availability of foods providing glucose (primarily as starch in grains) and fat, to be a significant contributor to increased obesity in the US.

## Abbreviations

HFCS: High fructose corn syrups; AUC: Area under the curve.

## Competing interests

Both authors declared that they have no competing interests.

## Authors’ contributions

TJC participated in study design, data collection and analysis, and drafted the manuscript. TPC conceived the study, participated in study design, and helped to draft the manuscript. Both authors read and approved the final manuscript.

## Supplementary Material

Additional file 1 List of 132 foods used to calculate USDA food availability. Click here for file
